# Hernia Causing Ureteral Obstruction With Hydronephrosis and Subsequent Urinary Tract Infection and Sepsis

**DOI:** 10.7759/cureus.29406

**Published:** 2022-09-21

**Authors:** Kevin D Healey, Ahmad O Rifai, Alexander J Maqueira, William Kantrales, Courtney Albury

**Affiliations:** 1 Urology, Lake Erie College of Osteopathic Medicine, Bradenton, USA; 2 Nephrology, The Virtual Nephrologist, Lynn Haven, USA; 3 Emergency Medicine, Orange Park Medical Center, Jacksonville, USA; 4 Medicine, Merit Health Wesley, Hattiesburg, USA

**Keywords:** sepsis, obstructive uropathy, inguinal hernias, hydronephrosis, acute kidney injury

## Abstract

Inguinal hernias are common anatomic defects, especially among men. Complications of inguinal hernias include incarceration, but incarcerated hernias rarely cause other disturbances. An 86-year-old man with a history of chronic kidney disease (stage IIIb) presented with recurrent urinary tract infections and acute kidney injury with sepsis. Physical examination revealed a right inguinal hernia, and non-contrast abdominal computed tomography revealed right ureteral obstruction and distal entrapment by the inguinal hernia, with hydronephrosis. The patient underwent right retrograde pyelography and ureteral stent placement, followed by laparoscopic inguinal herniorrhaphy with mesh, which restored renal function. Our case is unique among obstructive uropathies due to inguinal hernias because the distal ureter was entrapped within the bowel mesentery communicating between the peritoneal cavity and retroperitoneum. Both inguinal hernias and obstructive uropathy are common among elderly men. While the latter is often secondary to prostate malfunction, other causes of obstructive uropathy or hydronephrosis should be considered, especially if unilateral.

## Introduction

Inguinal hernias account for approximately 75% of all hernias diagnosed [1]. Over one million abdominal wall herniorrhaphies are performed annually in the United States, and approximately 800,000 of these are inguinal hernia repairs [1]. The incidence of inguinal hernia is higher in men (lifetime risk of 27%) than in women (3% lifetime risk). Furthermore, inguinal hernia incidence follows a bimodal age distribution, with the peaks occurring at approximately five and 75 years [2].

Risk factors for inguinal hernia development include excessive physical exertion, obesity, increasing age, and congenital anomalies such as patency of the processus vaginalis. While hernias can occur in multiple anatomical locations, they mainly involve the expulsion of intra-abdominal contents through a fascial defect. Hernial contents normally comprise sections of the small bowel and adipose tissue, which, on entrapment, can lead to complications such as bowel obstruction or strangulation [1]. These severe complications can be detected and repaired swiftly. In some instances, the ureter may become entrapped in the hernia sac, causing a ureteral obstruction that resolves after hernial repair [3]. Herein, we describe the case of an 86-year-old man who initially presented with sepsis secondary to a urinary tract infection (UTI). Our case is rare among previously reported cases of ureter entrapment within the hernia sac, as our patient’s ureter was compressed by the hernia sac, which is anatomically inexplicable due to the retroperitoneal location of the ureter.

## Case presentation

An 86-year-old man, with a one-week history of generalized weakness, malaise, and anorexia, developed chills and sweats that prompted him to visit the emergency room. He experienced increased urinary urgency and frequency with dysuria but denied hematuria. His past medical history included chronic kidney disease (CKD, stage IIIb), systemic hypertension, diabetes mellitus type II, and hyperlipidemia. Physical examination revealed a body temperature of 39.5 °C, heart rate of 120 beats/minute, and initial blood pressure of 147/76 mmHg. He appeared chronically ill and toxic, with dry mucous membranes. Lung examination was clear, and he had tachycardia without murmurs. Abdominal examination revealed diffuse nonspecific tenderness, more pronounced in the right lower quadrant. Further examination revealed a mass in the right inguinal area that was more pronounced on standing and coughing; hence, a right inguinal hernia was suspected. Initial blood work revealed leukocytosis with a left shift, elevated blood urea nitrogen (BUN), elevated creatinine and elevated lactic acid (Table 1).

**Table 1:** Initial blood test

Marker	Patient’s Value	Reference Value
White blood cell count	16.9 x 10^3^ cells/mm^3^	4.0 – 10 x 10^3^ cells/mm^3^
Neutrophils	93.6/HPF	42.2 – 75.2/HPF
Blood urea nitrogen (BUN)	65.0 mg/dL	6 – 21 mg/dL
Creatinine	1.8 mg/dL	0.5 – 1.2 mg/dL
Lactic Acid	2.8 mmol/L	0.3 – 1.5 mmol/L

 The patient was diagnosed with acute kidney injury (AKI). Urinalysis revealed pyuria, hematuria, nitrites, and moderate leukocyte levels, suggesting UTI (Table 2).

**Table 2:** Urinalysis results

Urinalyis		
White blood cells (WBC)	43 WBCs/HPF	0 – 2 WBCs/HPF
Red blood cells (RBC)	443 RBCs/HPF	42.2 – 75.2 RBCs/HPF
Nitrites	Positive	Negative
Leukocyte esterase	Positive	Negative

The patient was admitted for inpatient hospital care and ultimately diagnosed with sepsis. After appropriate blood and urine cultures were obtained, he received intravenous fluids of normal saline and 1 g of ceftriaxone intravenously.

**Figure 1:**
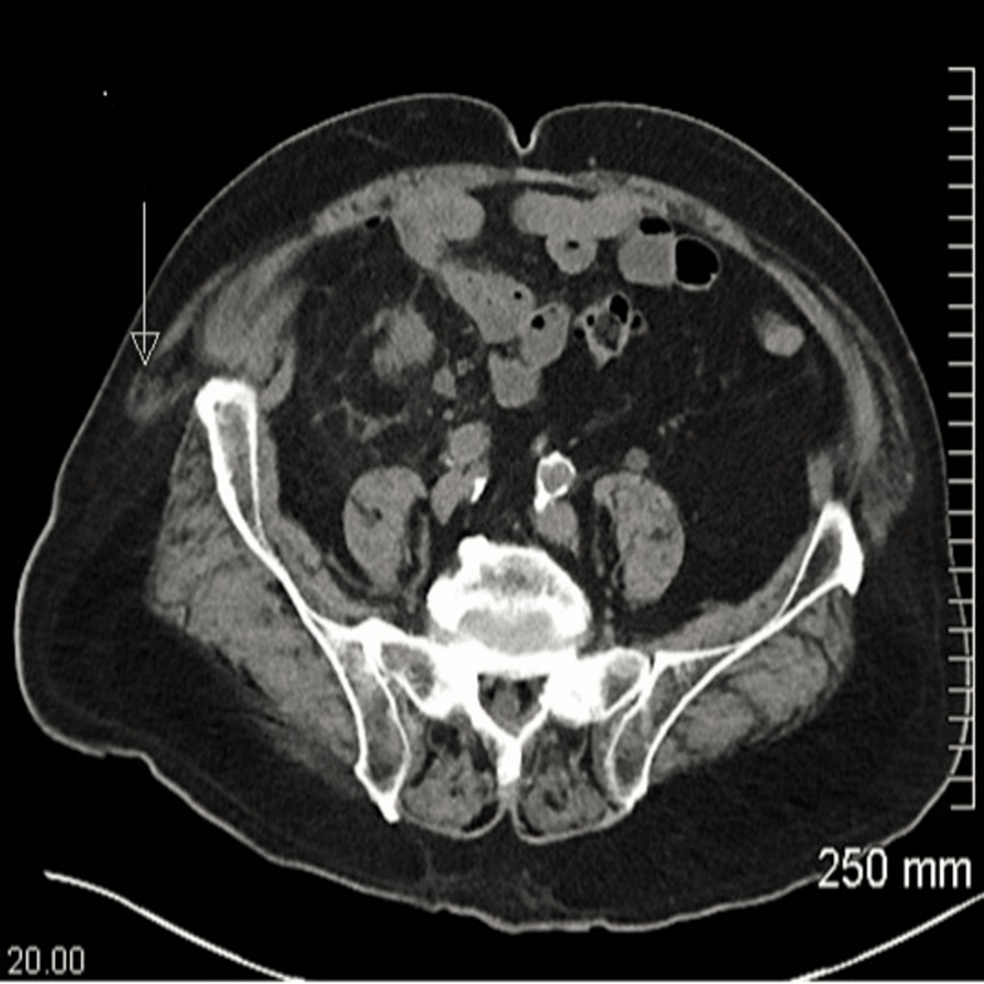
Computed tomography urogram without contrast with arrows pointing to the right distal ureter being compressed by the hernia sac

Renal ultrasound revealed mild hydronephrosis of the right kidney. A CT urogram revealed that the right distal ureter extended into the superior margin of the large right inguinal hernia, along with hernia-associated ureteral obstruction (Figure 1).

**Figure 2:**
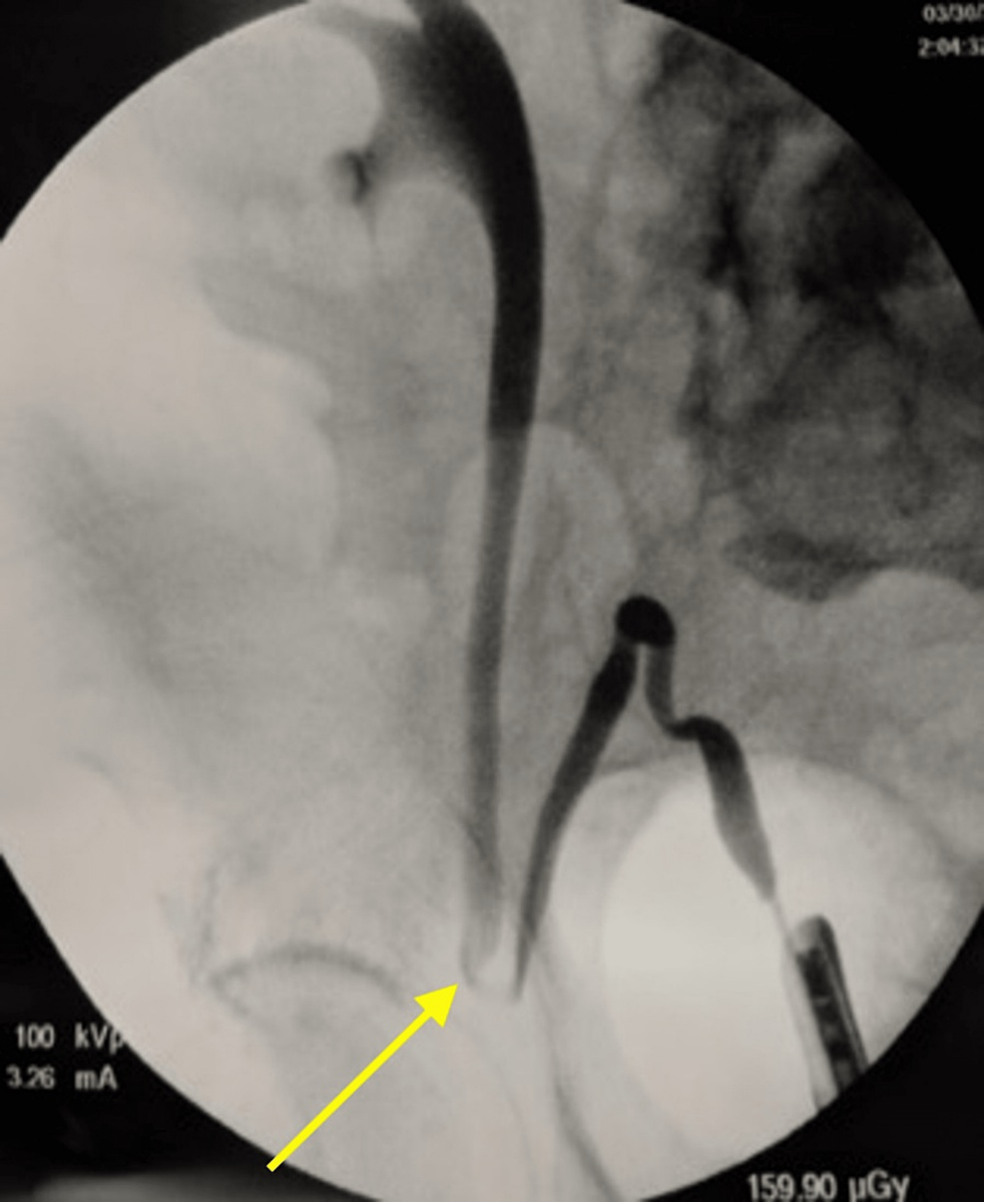
Retrograde ureterogram showing the compressed ureter

Retrogram ureterogram demonstrated significant distortion and obstruction of the distal ureter (Figure 2).

Our patient underwent cystoscopy with right retrograde pyelography and placement of a right 28×6-cm double-J ureteral stent. However, a prior attempt was made to place a Pollack catheter (Cook Medical Incorporated, Bloomington, Indiana) to gain access to the ureter and maintain its patency, followed by an attempt to place an Amplatz wire (Boston Scientific, Marlborough, Massachusetts) to guide the stent. These failed to straighten the ureter due to an extreme ureteric angulation. Fluoroscopic imaging demonstrated appropriate stent position.

Laparoscopic right inguinal herniorrhaphy was performed following stent placement. The hernia was repaired intraperitoneally with mesh placement, with no complications. The patient recovered with resolution of UTI, sepsis, and AKI, and the stent was removed.

## Discussion

We describe a rare case of a hernia compressing the ureter, resulting in ureteral obstruction and subsequent AKI, UTI, and sepsis. Normally, preoperative or intraoperative stent placement is unnecessary for hernia repair [4]; however, in our case, stent placement was necessary due to the unusual orientation of the ureter and its location relative to the hernia sac.

Abdominal hernias develop when the intra-abdominal contents protrude through a deficiency in the abdominal wall. There are several types of abdominal wall hernias, including inguinal, femoral, umbilical, epigastric, incisional, Spigelian, and parastomal. Approximately two-thirds of inguinal hernias are classified as indirect while one-third are classified as direct. Most childhood inguinal hernias are classified as indirect, occur lateral to the inferior epigastric vessels, and are thought to arise congenitally via patency of the processus vaginalis. Conversely, direct inguinal hernias are more common in older men, develop medial to the inferior epigastric vessels through Hesselbach’s triangle, and are thought to be related to the decreasing abdominal wall tissue strength that occurs with aging. Any chronic condition associated with increased intra-abdominal pressure may contribute to inguinal hernia development [5].

Many inguinal hernias are asymptomatic and detected only during a medical examination. Symptomatic patients often present with a painless groin swelling, which is more evident during upright and physical activities and characteristically resolves while recumbent. Often, the patient experiences groin pain, especially during strenuous physical activity or lifting. Occasionally, an inguinal hernia may become irreducibly incarcerated in the hernia sac. This may lead to increased groin pain and obstruction of the trapped bowel. Rarely (<3%), an inguinal hernia may become strangulated with a vascular compromise of the bowel [6]. The resulting intestinal ischemia can lead to perforation, peritonitis, and sepsis. The characteristic presentation may include a toxic-looking febrile patient with peritonitic signs on abdominal examination and a very tender irreducible inguinal hernia with overlying skin erythema. The morbidity of strangulated inguinal hernia is high, with a perioperative complication rate of 12% and a mortality rate of 6% [7].

Our case is unique because a direct inguinal hernia should not anatomically cause ureteral obstruction due to the specific locations of the structures involved, specifically the ureters residing in the retroperitoneum. Although there have been numerous cases of obstructive uropathy caused by ureteral inguinal hernias, the obstructive uropathy in our case was instead due to the unilateral external compression by the hernia sac on the ureter. In the only other reported case of ureteric compression by a hernia sac, the patient had an inguinoscrotal hernia that contained the colon, differing from the findings in our patient [3]. The distal ureter of our patient became entrapped within the mesentery at the communication point between the peritoneal cavity and retroperitoneum.

After the renal ultrasound showed mild right-sided hydronephrosis, the prompt CT urogram enabled visualization of the distal ureter extending into the inguinal hernial sac, resulting in ureteral obstruction and necessitating a surgical procedure. Most patients >60 years old have an increased risk of developing direct inguinal hernias, yet obstructive uropathy in this age group is primarily caused by benign prostatic hyperplasia [8]. However, in patients presenting with signs and symptoms of obstructive uropathy, UTIs, or unilateral hydronephrosis, it is important to consider and investigate other differential diagnoses, which may lead to an alternative treatment plan. 

## Conclusions

Obstructive uropathy due to inguinal hernias has previously been described as a result of incarceration. This unique case demonstrated distal ureter entrapment and compression within the bowel mesentery that communicated between the peritoneal cavity and retroperitoneum. This is an important, albeit rare, condition that general surgeons should keep in mind when investigating a hernia, even in patients without urinary symptoms.
